# Molecular Dynamics Study on Crack Propagation in Al Containing Mg–Si Clusters Formed during Natural Aging

**DOI:** 10.3390/ma16020883

**Published:** 2023-01-16

**Authors:** Sangjun Lee, Heon Kang, Donghyun Bae

**Affiliations:** 1 Department of Materials Science and Engineering, Yonsei University, Seoul 03722, Republic of Korea; 2 Heat & Surface Technology R&D Department, Korea Institute of Industrial Technology, Siheung 15014, Republic of Korea

**Keywords:** molecular dynamics, aluminum alloys, fracture, aging, clusters

## Abstract

The crack propagation behavior of Al containing Mg–Si clusters is investigated using molecular dynamics (MD) simulations to demonstrate the relationship between the natural aging time in Al–Si–Mg alloys and ductility. Experimental results show that the elongation at failure decreases with natural aging. There are few studies on the relationship between natural aging and ductility because of the difficult observation of Mg–Si clusters. To solve the difficulty, cracked Al containing Mg–Si clusters of varying sizes are assumed for the MD simulations. A larger Mg–Si cluster in Al results in earlier crack opening and dislocation emission. Moreover, as the Mg–Si cluster size increases, the stress near the crack tip becomes more concentrated. This causes rapid crack propagation, a similar effect to that of crack tip sharpening. As a result of long-term natural aging, the cracks expand rapidly. The influence of geometry is also investigated. Crack lengthening and thickness reduction negatively impact the fracture toughness, with the former having a larger impact than the latter. Although there are several discrepancies in the practical deformation conditions, the simulation results can help to more thoroughly understand natural aging in Al–Si–Mg alloys.

## 1. Introduction

Although the 6xxx series of Al alloys (Al–Si–Mg-based) have a high potential for application in automobile body parts due to their low density and high formability [1,2], there are still limitations due to their low ductility, as compared with ferrous alloys [3]. Natural aging in the 6xxx series of Al alloys is unavoidable due to a high concentration of vacancies [4,5,6]. Even at room temperature, rapid vacancy diffusion causes the formation of solute clusters, resulting in an undesirable increase in yield strength [7,8]. Therefore, the 6xxx series of Al alloys should be carefully managed after solution treatment to ensure their mechanical properties are maintained. Moreover, during artificial aging, several types of solute clusters formed during natural aging neither decompose nor transform into precipitates [8,9,10,11], resulting in low precipitate content after artificial aging. Controlling the solute clusters is necessary for the design of high-strength alloys because precipitate strengthening is one of the most significant strengthening mechanisms in the 6xxx series of Al alloys [12]. Pre-aging is commonly used to delay cluster formation during natural aging [13,14]. Because vacancies are rapidly annihilated in the typical artificial aging temperature range [15], short-term artificial aging immediately after solution treatment results in slow clustering. Many studies have been conducted to minimize the negative effects of natural aging by controlling the chemical composition of these Al alloys. Aruga et al. [8,9] demonstrated that Si-rich clusters are detrimental to the subsequent artificially aged properties; that is, the suppression of Si-rich cluster formation is required to achieve reasonable mechanical properties after artificial aging. Im et al. [16] investigated the bake-hardening response (an increase in yield strength during paint baking) in relation to the Mg/Si ratio. They reported that the bake hardening response depends on the natural aging time because Si-rich clusters predominate during the early stages of natural aging and Mg–Si clusters predominate at later stages. Despite extensive research on the strength of Al–Si–Mg-based alloys with respect to natural aging clusters, only a few studies on the influence of natural aging clusters on the ductility of these alloys have been performed.

Microcracks significantly impact the mechanical properties of Al alloys, particularly the ductility. Lados and Apelian [17] investigated the influence of grain size and Al–Si eutectic phase secondary dendrite arm spacing on crack propagation in cast Al–Si–Mg alloys. The effects of various alloying elements on the cracking behavior of Al–Si–Mg alloys, such as Mg [18] and Cu [19], were also analyzed. These studies, however, only focused on the relationship between micrometer-scale cracks and phases. Due to the excessively small size of the natural aging clusters (~200 atoms [4,9]), the crack propagation mechanism resulting from them has not been determined. Moreover, clusters grow even during specimen preparation or specimen observation owing to natural aging, making it difficult to determine an accurate relationship between the natural aging clusters and crack propagation mechanism. Molecular dynamics (MD) is a computer simulation method that calculates various physical properties at the atomic level [20]. It is useful for interpreting phenomena that are difficult to analyze using experimental methods. Several MD simulations of crack propagation in various metals, including Al [21,22,23], Fe [24], Mg [25], Ni [26], Cu [21,27], Ti [28], and metal matrices with various defect conditions [22,29,30,31], have been performed. Despite extensive research on crack propagation in metals, the cracking mechanisms of Al matrices containing Mg–Si clusters has not yet been investigated.

In this study, the influence of natural aging on crack propagation in Al was investigated using MD simulations. The motivation for this study was the experimental results that show a decrease in the elongation at failure with respect to natural aging time. Considering that natural aging in the 6xxx series of Al alloy is an unavoidable phenomenon and significantly affects formability, extensive studies on the relationship between natural aging and ductility are required. However, there are few studies on the impact of natural aging owing to the difficulty in observing Mg–Si clusters [32]. Therefore, MD simulation of crack propagation was considered. It is assumed that Mg–Si clusters of various sizes exist near the crack tip. A tensile test was simulated to determine the atomic-scale mechanisms of crack propagation. Demonstration of the detailed mechanism of crack propagation contributes not only to obtaining a high ductility 6xxx series of Al alloys but also to their application in industrial fields.

## 2. Experimental Details

The investigated Al alloy was cast using conventional gravity casting methods. The chemical composition of the investigated alloy was targeted to be similar to that of the commercial AA6016 alloy (Al–1.2Si–0.4Mg–0.3Cu–0.05Fe–0.05Mn–0.05Cr (in wt.%)) [33]. Pure Al was melted in a SiC crucible at 1033 K, and then Si, Mg, Cu, Fe, Mn, and Cr were added as alloying elements. To prepare an ingot, the melt was briefly stirred, maintained at temperature for 2 h, and then poured into a steel mold preheated to 523 K. The ingot was homogenized at 813 K for 12 h before cold rolling to a 1 mm thickness with a reduction ratio of 10% per pass. After cold rolling, the investigated alloy sheet was machined to a gauge length of 25 mm according to the ASTM E8 standard to prepare tensile testing specimens. After 30 min of solution treatment at 813 K, the specimens were quenched with water. Tensile tests were performed at a strain rate of 10^−3^ s^−1^ at room temperature (293 K). The tensile tests were repeated 5 times for each NA condition to ensure reproducibility.

## 3. Molecular Dynamics (MD) Simulation

### 3.1. Interatomic Potential

The overall system was demonstrated using a hybrid pair style with distinguishable interatomic potentials. Nandy et al. [34] used MD simulations to analyze AlSi10Mg particles using a combination of the embedded atomic method (EAM) and the Tersoff and Lennard–Jones (LJ) potentials. The EAM potential was used to describe the Al–Al [35] and Mg–Mg [36] pair potentials, and the total energy *E* of *N* atoms can be expressed as follows [35,36,37]:

(1) $$E=\overset{N}{\underset{i=1}{\sum }}F_{i}\left(\rho_{i}\right)+\frac{1}{2}\sum_{i,j}^{N}\phi_{ij}\left(r_{ij}\right)$$

where *F_i_* represents the embedding energy function of an atom *i* for the electron density function, and *ρ_i_* and *ϕ_ij_* represents the repulsive pair potential between atoms *i* and *j* with a distance *r_ij_*. The Si–Si [38] pair potential was described using the Tersoff potential, and the potential energy *E* can be expressed as [38,39]:

(2) $$E=\frac{1}{2}\underset{i}{\sum }\underset{j\neq 1}{\sum }V_{ij}$$

where *V_ij_* represents the bonding energy between atoms *i* and *j*. The LJ potential was used to describe heterogeneous pair interactions (Al–Si, Al–Mg, and Mg–Si pairs). The potential energy *U* in relation to the atomic distance *r* can be expressed as follows [40]:

(3) $$U\left(r\right)=4\varepsilon \left[{\left(\frac{\sigma }{r}\right)}^{12}-{\left(\frac{\sigma }{r}\right)}^{6}\right]$$

where *ε* and *σ* represent the lowest energy of the potential curve and the interatomic distance at which the potential energy is zero, respectively. Table 1 lists the parameters required to calculate the LJ potential.

### 3.2. Atomistic Modeling

**Figure 1:**
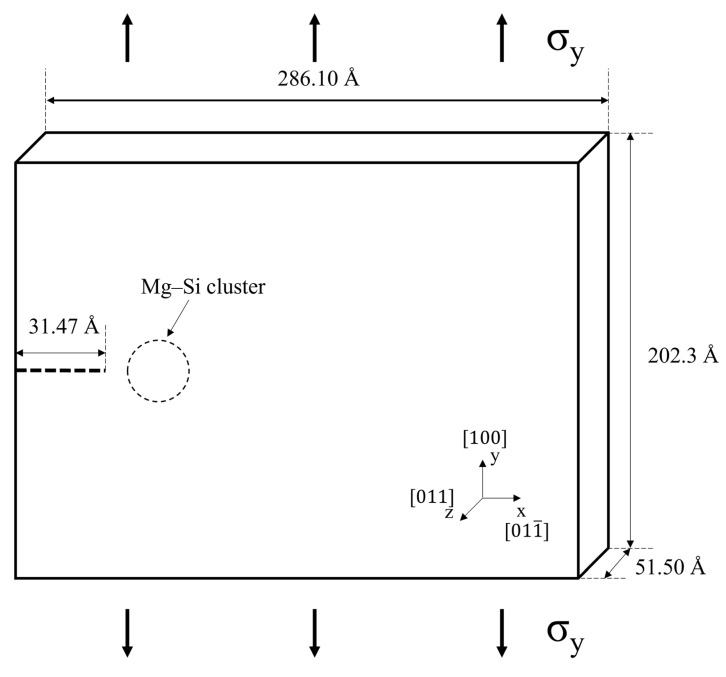
Schematic representation of the MD simulation modeling.

The crack propagation simulation was performed based on MD simulations using the large-scale atomic/molecular massively parallel simulator (LAMMPS) software created by Sandia National Laboratories [41]. Figure 1 schematically depicts the model used to analyze crack propagation. The x, y, and z axes of the Al face-centered cubic (FCC) lattice was oriented along the [01$\bar{1}$], [100], and [011] directions, respectively. Because mobile dislocations obeying the FCC slip system are perpendicular to the xy plane, this combination of orientations was selected to facilitate dislocation observation. The size of the Al supercell was 50 × 50 × 9 unit cells (286.10 × 202.30 × 51.50 Å). The x- and y-axis boundaries were set to be free, whereas the z-axis boundary was set as periodic. To avoid boundary effects, the atoms in the outermost unit cell of the y-axis boundary were made rigid. An x-axis-elongated crack (length: 31.47 Å, width: 8.092 Å) with a sharp tip (angle to x-axis: 35.26°) was assumed to exist at the center of the yz-plane. Spherical Mg–Si clusters with radii of 4, 6, 8, and 9 Å were also assumed to exist 10 Å away from the crack tip. The given radii were selected to avoid interaction between the Mg–Si clusters and the atoms outside the simulation region, considering the cutoff radius for the LJ potential and the selected dimension for simulating the area thickness. Because Mg–Si clusters are formed by vacancy diffusion, the crystal structure of the Mg–Si cluster was assumed to be FCC with an interface that is perfectly coherent with the Al matrix [42]. The Mg–Si clusters with radii of 4, 6, 8, and 9 Å were composed of 15, 55, 130, and 189 solute atoms, respectively, according to the assumptions used to build the Mg–Si cluster. Mg–Si clusters containing 15 and 189 solute atoms are comparable in size during the early [10] and sufficiently late (~1 year [9]) stages of natural aging in Al–Si–Mg alloys. The chemical composition of the Mg–Si cluster was selected to be Mg/(Mg + Si) = 0.5, which is the most stable for subsequent artificial aging [43]. Because the number of considered solute atoms in the Mg–Si clusters having radii of 4, 6, and 9 Å is odd, the composition cannot be Mg/(Mg + Si) = 0.5. Mg atoms were assumed to be more abundant than Si atoms in these cases, and the differences in the Mg–Si cluster with the composition Mg/(Mg + Si) = 0.5 were assumed to be negligible. The atomic model was initially equilibrated at 0 K for 1 ps using the Nosé–Hoover thermostat [20]. At 0 K, velocity loading with a strain rate of 10^10^ s^−1^ was applied on the top and bottom boundaries along the y-axis without controlling the heat generated during tensile loading. Despite the unrealistic deformation conditions, many researchers have conducted several MD tensile deformation simulations under the given conditions [21,22,23,24,25,26,27,28,29,30,31]. The timestep was set to 0.001 ps in all MD simulations.

### 3.3. Numerical Algorithm for Solving MD Equations

The atomic stress *σ* was calculated using the Virial theorem, which can be expressed as follows [26,44]:

(4) $$\sigma_{\alpha \beta }=\frac{1}{\Omega }\overset{N}{\underset{i=1}{\sum }}\left(-m^{i}\left(v_{\alpha }^{i}-\bar{v}\right)\left(v_{\beta }^{i}-\bar{v}\right)+\frac{1}{2}\underset{j\neq 1}{\sum }\left(x_{\alpha }^{j}-x_{\beta }^{j}\right)F_{\beta }^{ij}\right)$$

where *α* and *β* denote the specific direction of the stress tensor; *Ω* denotes the volume of the simulation area; *i* and *j* denote specific atoms; *N* denotes the total number of atoms; and *F^ij^* denotes the force on atoms *i* and *j* resulting from the pairwise interaction. The LAMMPS software includes a calculation package for calculating the stress per atom. The tensor for each atom in a cubic system is symmetric, with only six components. Because tensile loading was applied along the y-axis, the primary focus of this study was *σ_yy_*. The strain *ε* was calculated as follows [23]:

(5) $$\varepsilon =\frac{L-L_{0}}{L_{0}}$$

where *L* and *L*_0_ represent the y-axis lengths of the simulation area at a specific step and initial step, respectively.

Understanding the atomic scale deformation mechanism requires analyzing the crystal structures (for example, dislocations and twins) that correspond to each atom. The atomic configurations were visualized using the OVITO software, which provides a dislocation analysis tool based on center-symmetry (CSYM) parameters. The CSYM parameter *ρ_i_* of an atom *i* can be calculated as follows [23,26,45]:

(6) $$\rho_{i}=\overset{6}{\underset{i=1}{\sum }}{\left|{\vec{R}}_{i}+{\vec{R}}_{i+6}\right|}^{2}$$

where ${\vec{R}}_{i}$ and ${\vec{R}}_{i+6}$ are six pairs of position vectors of the relative nearest atoms in the FCC structure. In the FCC structure, the CSYM parameter exhibits a value of 0–12, with 3–9 being considered a dislocation [23].

## 4. Results

**Figure 2:**
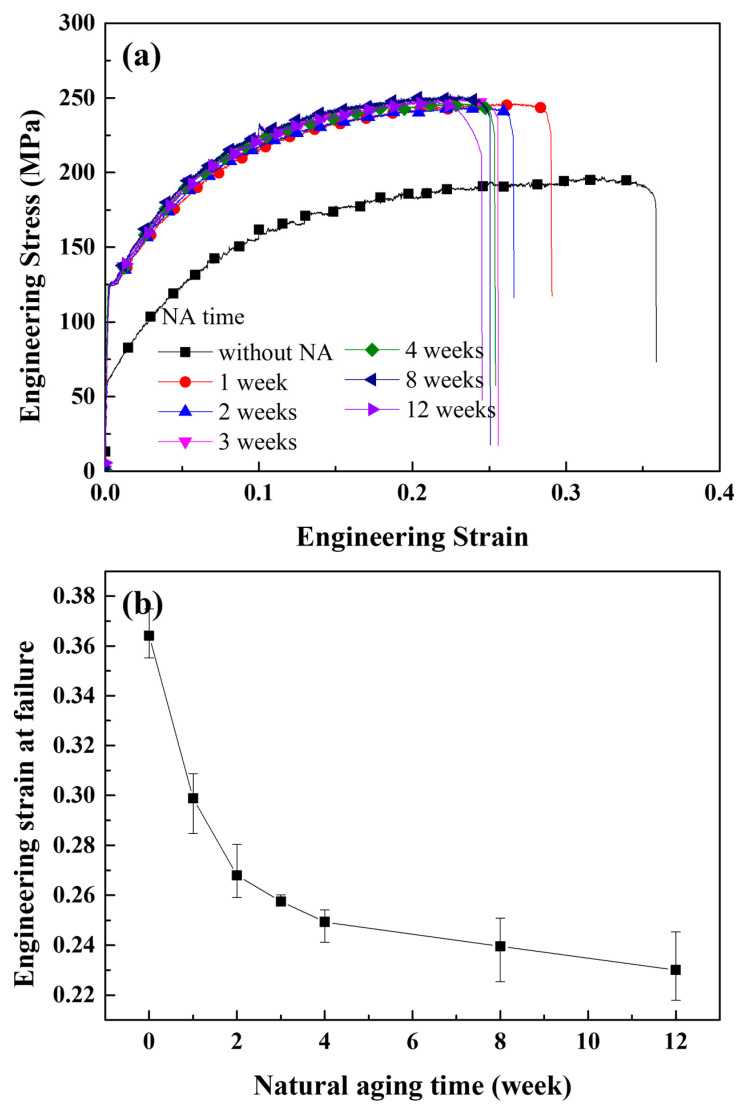
Experimentally obtained mechanical properties of the investigated Al–Si–Mg-based alloy. (**a**) Engineering stress-strain curve with various natural aging times and (**b**) engineering strain at failure as a function of the natural aging time.

**Figure 3:**
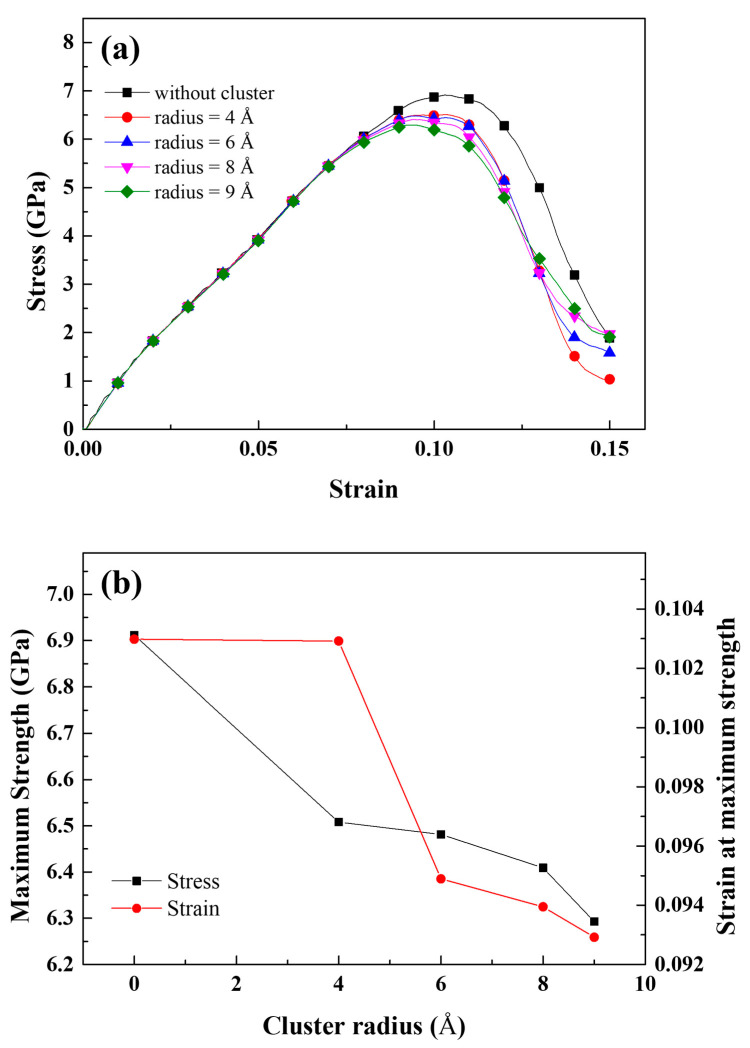
(**a**) Stress–strain curve of a cracked Al single crystal containing Mg–Si clusters with radii of 0, 4, 6, 8, and 9 Å. (**b**) Maximum stress and the strain at maximum stress as a function of the cluster radius.

Figure 2a shows the tensile stress–strain curves for various natural aging times. The yield strength value when natural aging did not occur was the lowest of all the conditions tested. Only one week of natural aging resulted in a significant yield strength increase. The yield strength after more than one week of natural aging showed a negligible difference from that of the sample aged for one week. However, the ductility change as a result of natural aging was the primary focus of this study. Figure 2b depicts the engineering strain at failure in relation to the natural aging time, which indicates the ductility of the samples. The highest elongation at failure was observed when natural aging had not occurred and gradually decreased as natural aging progressed. Due to the interaction between dislocations and Mg–Si clusters, the elongation at failure rapidly decreases in the early stages of natural aging [46], which is indicated by the increase in yield strength. Following two weeks of natural aging, the rate of decrease significantly slowed. This phenomenon may be related to the rate at which Mg–Si clusters grow. As previously reported, the growth rate of Mg–Si clusters gradually slows as natural aging progresses, as inferred from the mechanical or electrical properties [6,7,8] and kinetic Monte Carlo simulation results [43,47]. However, the typical size of Mg–Si clusters is extremely small and could insignificantly increase the interaction between dislocations and Mg–Si clusters. Therefore, another embrittlement mechanism should be discussed, and the effect of Mg–Si clusters on crack propagation should be considered. The size of the Mg–Si clusters increased as the natural aging time increased. We previously proposed that Mg–Si cluster growth, rather than nucleation, predominantly occurs after 30 min of natural aging [43]. That is, the effect of the gradually increasing Mg–Si cluster size reflects the effects of the natural aging time. After only two days of natural aging, Fallah et al. [10] discovered ~30 atoms that were larger than the 4 Å of the Mg–Si cluster. After one year of natural aging, Aruga et al. [9] discovered ~200 atoms in Mg–Si clusters. According to the experimental results, the given radius conditions could be considered as the practical natural aging conditions from the early stage to the sufficiently late stage. Figure 3a shows the stress–strain curve of an Al single crystal containing Mg–Si clusters with radii of 4, 6, 8, and 9 Å as obtained from the MD simulations. The overall trends for each curve were similar. The maximum stress decreased as the Mg–Si cluster radius increased, whereas the strain at the maximum stress showed the opposite pattern, as shown in Figure 3b.

**Figure 4:**
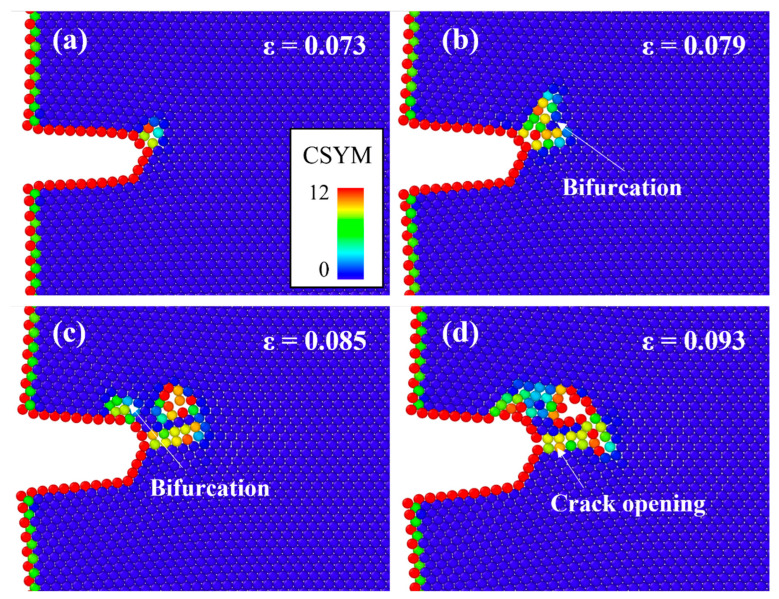
Atomic configurations of a cracked Al single crystal at the strain of (**a**) *ε* = 0.073, (**b**) *ε* = 0.079, (**c**) *ε* = 0.085, and (**d**) *ε* = 0.093. The different colors represent the CSYM parameters.

Figure 4 depicts the atomic configurations of Al without Mg–Si clusters at specific strains during tensile deformation. The various colors represent the CSYM parameters used to determine the change in the crystal structure. During the early stages of deformation, Al deforms with a constant volume, and the CSYM parameters of the atoms near the crack tip begin to change at *ε* = 0.073 (Figure 4a). The change in the CSYM parameters propagates along the (11$\bar{1}$) plane, which is a representative FCC slip plane and starts to bifurcate at *ε* = 0.078 (Figure 4b). Although the CSYM parameter changed, dislocations could not be considered because the magnitude of the CSYM parameters change was irregular. A half-plane insertion with regular CSYM parameters can be used to determine the dislocations. The crack tip began to open at *ε* = 0.093 and the crack propagated (Figure 4d). As the tensile deformation progressed, micropores were observed near the crack tip and joined the crack propagation line. The micropores, formed as a result of the agglomeration of several vacancies, blunted the crack tip and slowed crack propagation [21]. No dislocation emission from the crack tip was observed during the tensile deformation [21].

**Figure 5:**
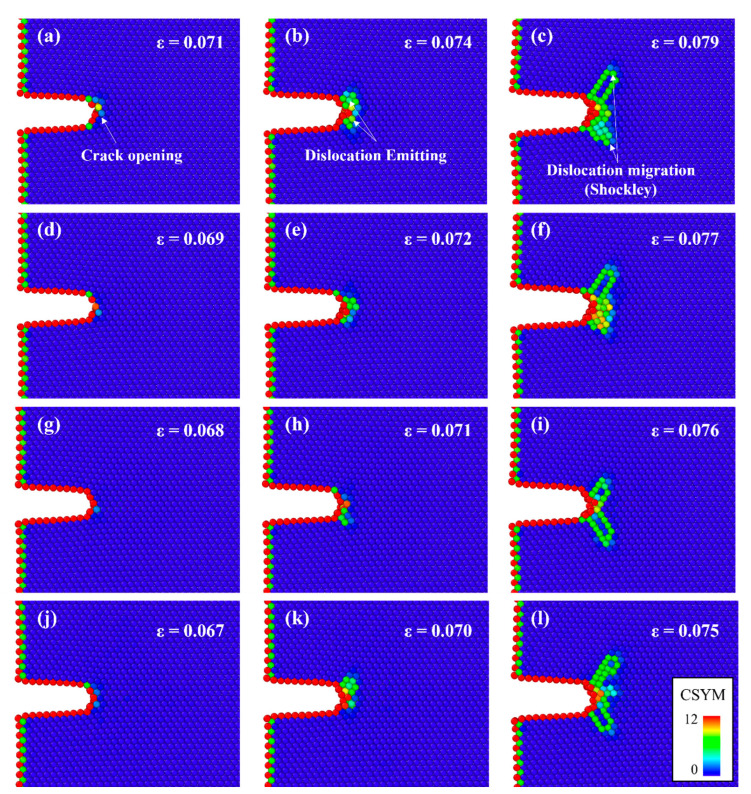
Atomic configurations of the cracked Al single crystal containing Mg–Si clusters with the radii of (**a**–**c**) 4, (**d**–**f**) 6, (**g**–**i**) 8, and (**j**–**l**) 9 Å. The different colors represent the CSYM parameters. The left column (**a**,**d**,**g**,**j**) shows the crack-opening strains; the middle column (**b**,**e**,**h**,**k**) shows the dislocation emitting strains; and the right column (**c**,**f**,**i**,**l**) shows the strain where Shockley dislocations sufficiently migrate.

**Figure 6:**
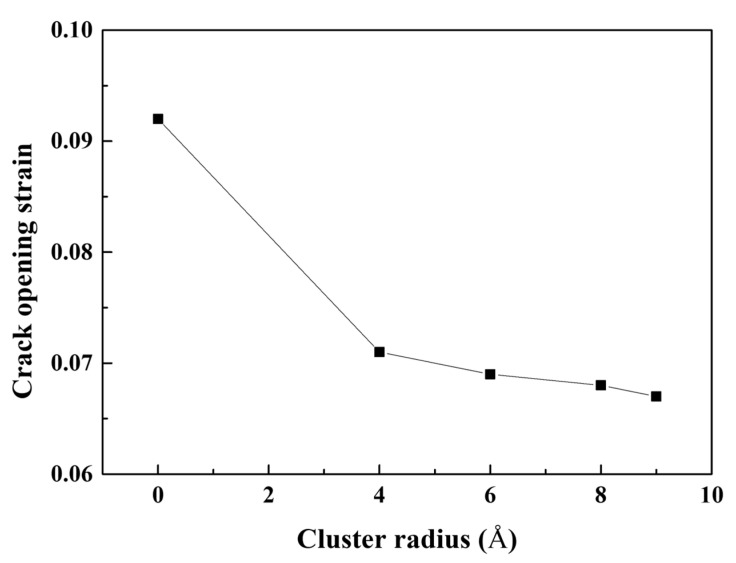
Crack-opening strains of the cracked Al containing an Mg–Si cluster as a function of the Mg–Si cluster radius.

Figure 5 depicts the atomic configurations of Al containing Mg–Si clusters with radii of 4, 6, 8, and 9 Å in terms of the CSYM parameters. The strains at which the crack tips start to open were significantly lower for Al containing Mg–Si clusters of varying radii than for Al without Mg–Si clusters. Notably, regardless of the radius of the Mg–Si cluster, dislocations were continuously emitted after the crack opened. The emitted dislocations migrated as the tensile deformation progressed and were confirmed to be $\frac{1}{6}${111}〈112〉 Shockley partial dislocations. Perfect dislocations were not emitted until the tensile deformation was complete. This is due to a decrease in the energy of the localized stacking fault near the Mg–Si cluster [48]. For each cluster radius condition, the dislocation emission strain was 0.003 greater than the crack-opening strain. Figure 6 shows a plot of the crack-opening strain as a function of the Mg–Si cluster radius. As the radius of the Mg–Si cluster increased, the strains at which the crack began to open and dislocations were emitted gradually decreased. Although the size of the Mg–Si cluster affects the crack-opening strain, the effect of the Mg–Si cluster size is less than that of the presence of Mg–Si clusters. The experimentally obtained strains at failure followed a similar pattern. As shown in Figure 2, the engineering strain at failure abruptly decreased when Mg–Si clusters were formed and gradually decreased with an increasing Mg–Si cluster size.

**Figure 7:**
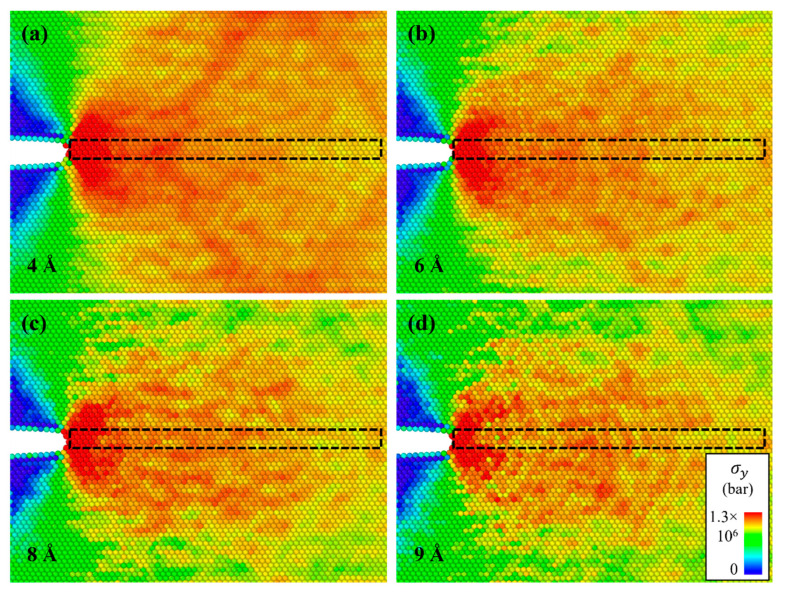
Atomic configurations of the cracked Al containing an Mg–Si cluster with a radius of (**a**) 4, (**b**) 6, (**c**) 8, and (**d**) 9 Å. The configurations were snapshots of their crack-opening strains. The different colors represent the y-axis normal stress in the tensile loading condition. The black dotted box represents the atoms along the crack propagation lines (in this case, parallel to the x-axis). The quantitative stress of the atoms in the marked box is shown in Figure 8.

**Figure 8:**
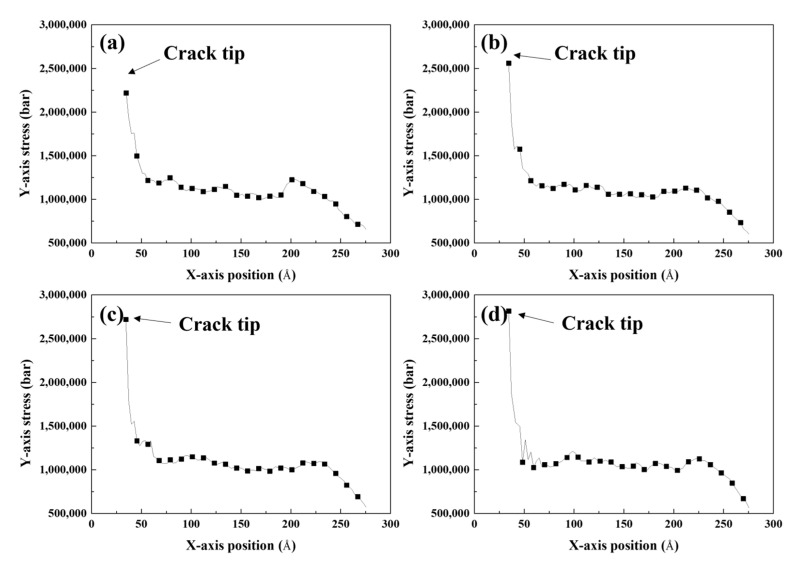
Average y-axis normal stress of the cracked Al containing an Mg–Si cluster with the radius of (**a**) 4, (**b**) 6, (**c**) 8, and (**d**) 9 Å as a function of the x-axis position (parallel to the crack direction). The atoms involved in this calculation were positioned along the crack direction (the region demarcated by the dotted box in Figure 7). Each calculation was conducted at the crack-opening strain.

Figure 7 shows the atomic configurations of the cracked Al with Mg–Si clusters at each crack-opening strain. The various colors represent the y-axis normal stress per atom. For every Mg–Si cluster radius, the atoms near the crack tip exhibited higher stress than the atoms far from the crack tip. This is known as stress concentration [49,50], which results in crack propagation along the crack tip line. The stress in specific areas varied as *x*^0.5^ with the distance from the crack tip [50]. The stress per atom decreased as the distance from the crack tip increased for atoms along the crack direction. Figure 8 depicts the relationship between the x-axis position and y-axis normal stress for the atoms highlighted in the black dotted box in Figure 7. The stress at the atoms far from the crack tip decreased, reflecting the stress concentration. The curves exhibit inflection points that defy the established relationship [49], which could be due to the atom positions that are far from the crack tip. The stress components describing the fracture situation are only defined near the crack tip.

**Figure 9:**
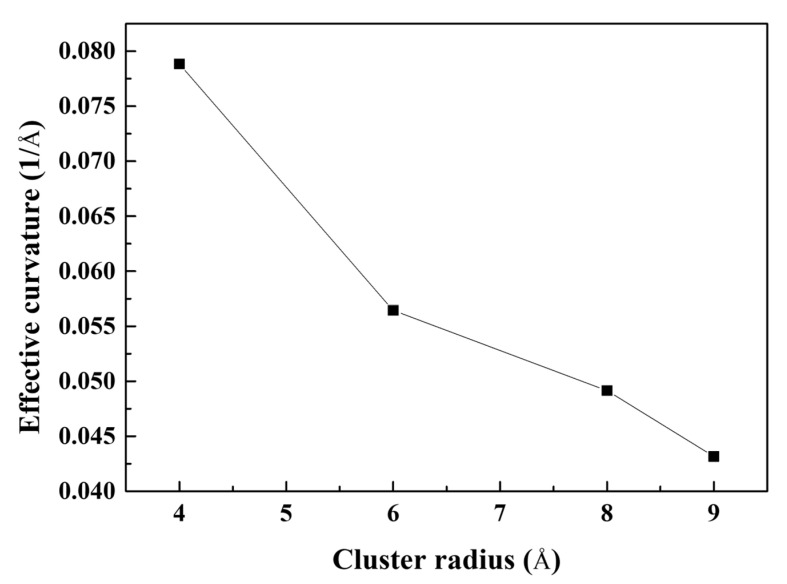
Effective curvature of the cracked Al containing Mg–Si clusters as a function of the cluster radius.

**Figure 10:**
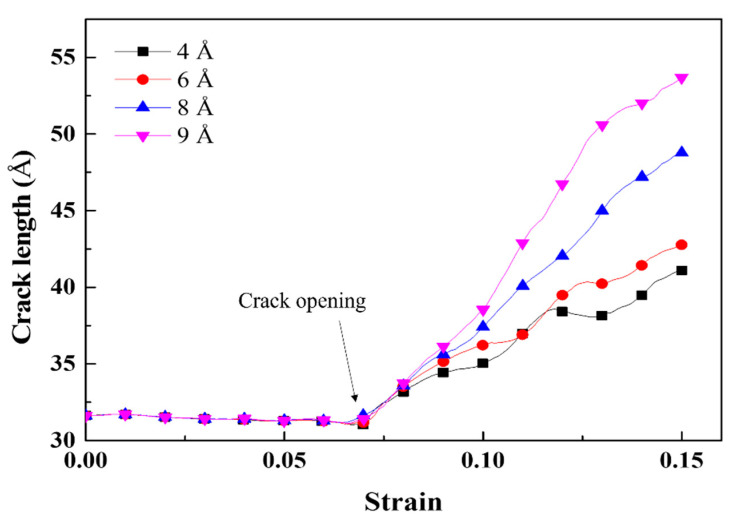
Crack length of the cracked Al containing Mg–Si clusters with radii of 4, 6, 8, and 9 Å as a function of the strain. The lengthening of the crack indicates crack propagation.

The y-axis normal stress at the crack tip (maximum stress) decreased as the radius of the Mg–Si cluster increased. The stress concentration factor *K*, which can be defined as the ratio of stress in a specific region to the nominally applied stress, is commonly used to explain fracture behavior. The magnitude of stress concentration in an ellipsoid crack is determined by the curvature of the crack tip. It was difficult to determine the curvature of the crack because the crack tip was assumed to be sharp. To solve this problem, the following relationship between stress concentration and geometrical factors was considered [49]:

(7) $$\sigma_{max}≅2\sigma_{nom}{\left(\frac{c}{\rho }\right)}^{1/2}$$

where *σ_max_*, *σ_nom_*, *c*, and *ρ* represent the maximum stress, nominal applied stress, crack length, and radius of curvature of the crack tip, with *ρ* ≪ *c*, respectively. Because the theoretical radius of the curvature of a sharp crack tip is zero, the calculated results for *ρ* were regarded as the effective curvature. Figure 9 shows the effective curvature of the crack tip as a function of the Mg–Si cluster radius. The effective curvature of the crack tip decreased with an increasing Mg–Si cluster radius, as indicated by the decreasing trend of the maximum stress in Figure 8. This indicates that the growth of the Mg–Si cluster exhibited a similar effect to crack tip sharpening, which is detrimental to crack propagation. Figure 10 depicts the crack propagation in the pre-cracked Al containing Mg–Si clusters with radii of 4, 6, 8, and 9 Å as a function of the strain. The crack length was determined as the distance between the crack tip and the most distant atom. The crack length was maintained with negligible errors for each radius of the Mg–Si cluster until the crack-opening strain was reached. The crack length increased significantly after the crack-opening strain was reached, indicating crack propagation. When the radius of the Mg–Si cluster was varied, the crack propagation rate after the crack-opening strain was reached significantly differed. The larger the radius of the Mg–Si cluster, the faster the crack propagated.

**Figure 11:**
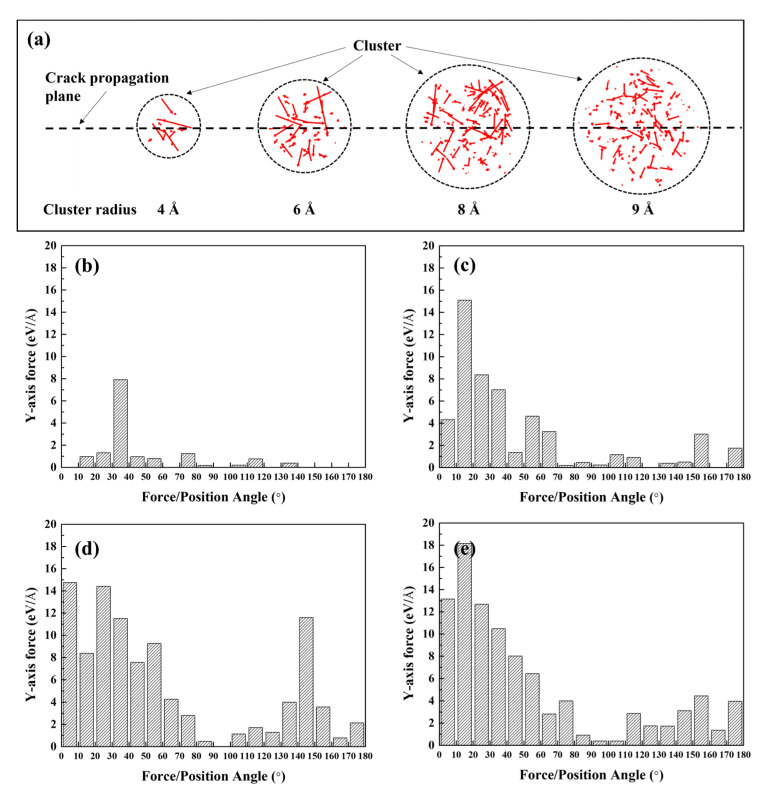
(**a**) Force vector plot of the cracked Al containing Mg–Si clusters with radii of 4, 6, 8, and 9 Å. Each figure was rendered in a pre-deformed state. The black dotted circles and red arrows represent the Mg–Si cluster and force vectors applied to each solute atom, respectively. Solute atoms are not visible. (**b**–**e**) Distribution of the y-axis force as a function of the angle between the force vector and crack plane. Each figure was reconstructed using the vector plot of the pre-deformed Al containing Mg–Si clusters with radii of (**b**) 4, (**c**) 6, (**d**) 8, and (**e**) 9 Å, which are shown in (**a**).

The force applied to the Si and Mg solute atoms may cause a crack-tip sharpening effect. Figure 11a shows a vector plot of the applied force per solute atom in the pre-deformed state. The magnitude of the forces applied to Al atoms was significantly smaller than those applied to Mg and Si, indicating that the force contribution of Al was negligible. The force vector tended to be directed toward the crack propagation plane, indicating that the crack tip morphology was considered to be flatter and sharper. Figure 11b–e quantitatively depicts the tendency of the force vector direction; that is, the figure depicts the y-axis force on the solute atoms in relation to the angle formed by the force vector and crack plane. Forces with angles of less than 90° can be categorized as crack-thinning forces. The force distribution indicates that at every cluster radius condition, the overall crack-thinning force was greater than the crack-thickening force, resulting in a sharpening of the effective curvature of the crack tip. Furthermore, as the number of solute atoms increased, the difference between the thinning and thickening forces also increased. Therefore, the growth of Mg–Si clusters aided rapid crack propagation, indicating embrittlement after natural aging.

## 5. Discussion

**Figure 12:**
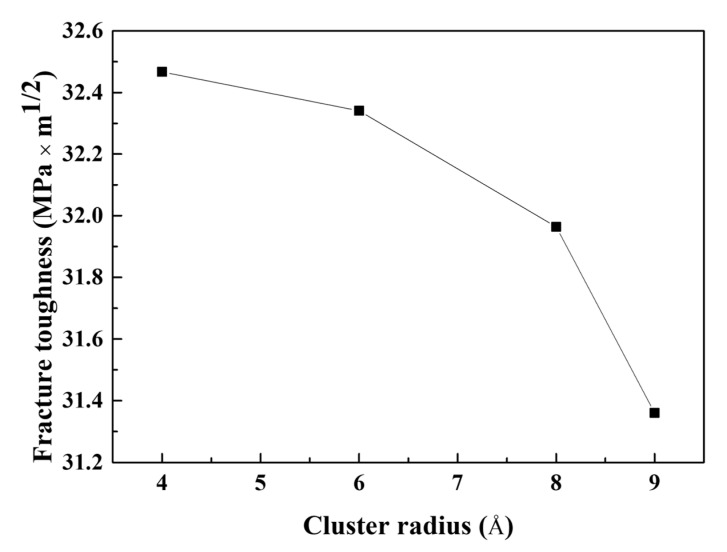
Fracture toughness of the cracked Al containing Mg–Si clusters with radii of 4, 6, 8, and 9 Å as a function of the cluster radius. The fracture toughness was calculated using the empirical equations typically used in experimental measurements.

Fracture toughness *K_1C_*, which indicates the physical resistance to crack propagation, is commonly regarded as an important factor when discussing fracture mechanics. Experimental test methods have been established for various specimen types [50]. In the most applicable experimental method for this simulation, the fracture toughness can be expressed as follows [50,51]:

(8) $$K_{1C}=\sigma_{F}\sqrt{\pi c}f\left(c/a\right)$$

(9) $$f\left(\frac{c}{a}\right)=\frac{1}{\pi }\left[29.6-185.5\left(\frac{c}{a}\right)+655.7{\left(\frac{c}{a}\right)}^{2}-1017{\left(\frac{c}{a}\right)}^{3}+638.9{\left(\frac{c}{a}\right)}^{4}\right]$$

where *σ_F_*, *a*, and *c* represent the nominal applied stress on the specimen, the length of the specimen, and the crack length, respectively. Figure 12 depicts the fracture toughness of the cracked Al containing Mg–Si clusters determined using experimental calculations. The fracture toughness values in this calculation were consistent with the values of conventional Al [52]. The fracture toughness decreased as the radius of the Mg–Si cluster increased. As shown in Section 4, large Mg–Si clusters cause rapid crack propagation.

**Figure 13:**
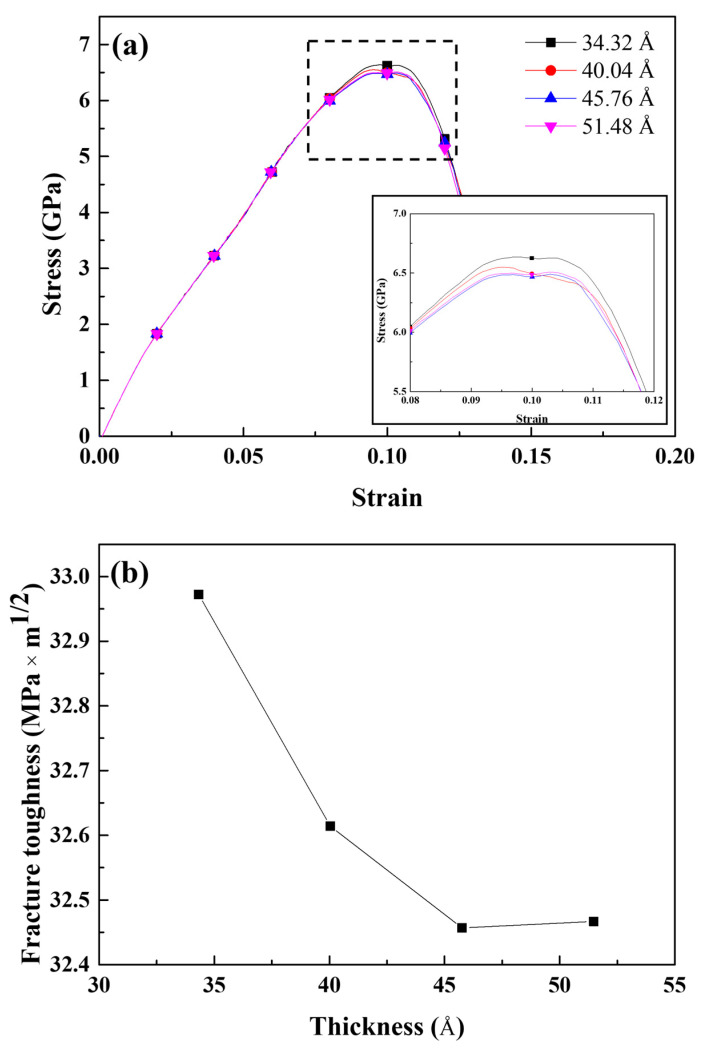
(**a**) Tensile stress–strain curves and (**b**) fracture toughness of the cracked Al containing 4 Å Mg–Si clusters with various crack lengths.

The experimental fracture toughness is affected by geometrical factors, one of which is the crack length, as confirmed by Equations (8) and (9). An atomic model similar to that shown in Figure 1 was developed with the crack lengthened to 40.05, 51.50, and 60.08 Å. The radius of the Mg–Si cluster was set to 4 Å. An MD simulation for tensile loading with identical loading conditions as described in Section 3 was performed. Figure 13a depicts the tensile stress–strain curves of the cracked Al containing Mg–Si clusters with varying crack lengths obtained from the MD simulation. The maximum stress and the strain at which the stress reaches its maximum value decreased significantly as the crack length increased. Furthermore, the crack-opening strain and dislocation-emitting strain decreased. The crack-opening strain and dislocation-emitting strain for the longest crack were 0.044 and 0.054, respectively, which were significantly lower than those in Figure 5a,b. This result is consistent with that of Cui and Beom [21]. They discovered a decreasing stress and crack-opening strain trend as the crack length increased, and Al containing Mg–Si clusters showed similar results but with dislocation emission. The fracture toughness, as shown in Figure 13b, can also address the effect of the crack length. Geometrical reasons indicate that fracture toughness is significantly reduced in relation to crack length. Under the assumption that the crack was elliptical, the curvature of the crack tip with an identical width decreased as the crack lengthened. As previously illustrated, this causes a crack tip sharpening effect, which facilitates rapid crack propagation. When the values in Figure 12 and Figure 13b are considered, the crack length contributes more to crack propagation than the Mg–Si cluster size.

**Figure 14:**
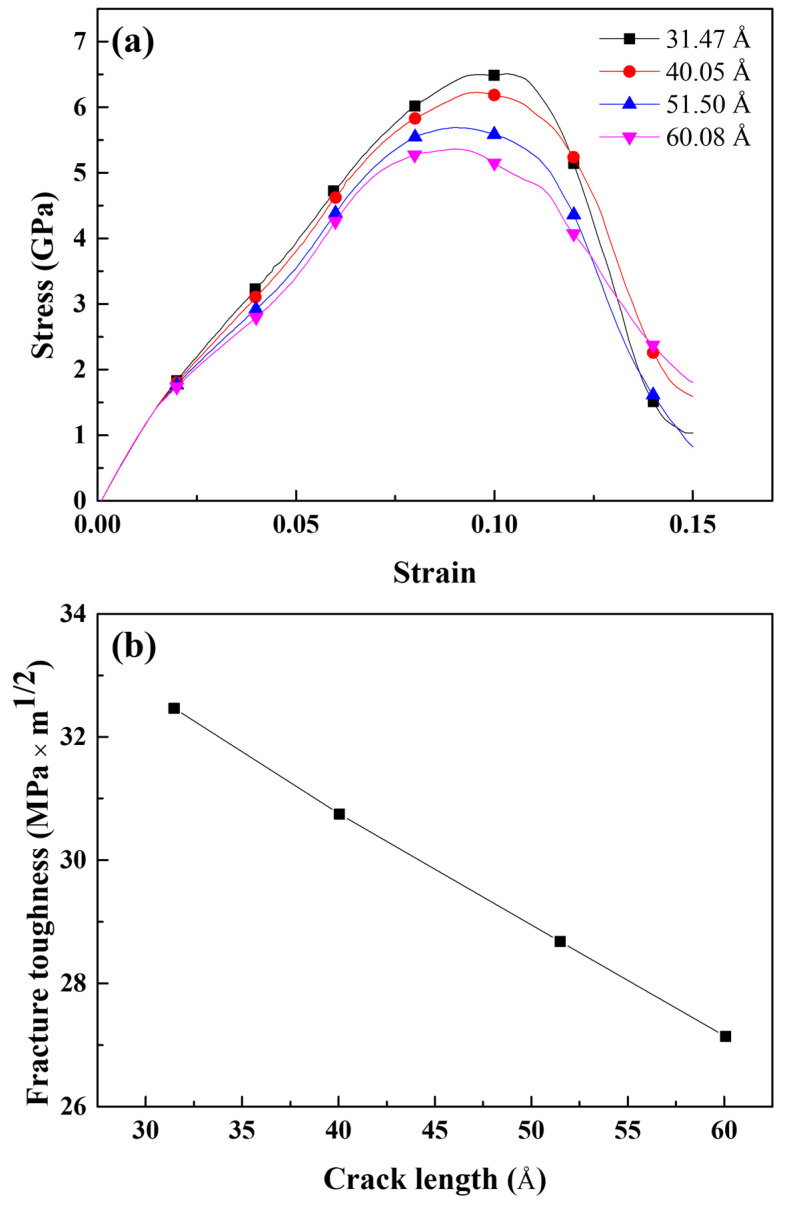
(**a**) Tensile stress–strain curves and (**b**) fracture toughness of the cracked Al containing 4 Å Mg–Si clusters with various thicknesses.

It is commonly assumed that fracture toughness decreases as the thickness of the specimen increases and eventually converges after a specific critical thickness [49,53]. MD simulations of tensile tests with identical conditions to those described in Section 3 but with thicknesses of 34.32, 40.04, and 45.76 Å were performed to demonstrate this phenomenon. Figure 14a depicts the tensile stress–strain curves of the cracked Al containing 4 Å Mg–Si clusters of varying thicknesses obtained from the MD simulation. The maximum stress decreased as the thickness increased, resulting in a decrease in the fracture toughness, as shown in Figure 14b. This pattern reflects the experimentally verified relationship between fracture toughness and specimen thickness. However, the magnitude of the decrease appears to be insignificant when compared with that observed in Figure 13b. Furthermore, the crack-opening strain, dislocation emitting strain, and crack propagation rate all varied slightly with thickness. The size effect should also be considered in MD simulations. Ding et al. investigated the size effect in MD simulations of cracked Al tensile tests. In a larger-scale simulation, they discovered that the yield stress was higher, and the crack propagated more rapidly. The magnitude of the size effects reported by Ding et al. differed from the results of this study. Many factors may contribute to this, including the total number of atoms in the simulation region, loading conditions, temperature, and geometry. Moreover, the simulation area was extremely small to draw any conclusions at the practical specimen scale. Although the results in Figure 14 show a similar trend to those of the experimental results, this phenomenon requires further discussion.

The elongation at failure is affected not only by the crack propagation behavior but also by other factors, such as the fabrication process [54,55,56,57], texture [58,59,60], and grain size [56,57,60]. This study only addresses the situation where the Mg–Si cluster is close to the crack; however, there could be significant Mg–Si clusters further away from the crack. Furthermore, the chemical composition of the alloy [11,16,43], vacancy concentration [43,47], and dislocation density [43] all influence the growth rate of Mg–Si clusters. The tensile test was performed using an MD simulation at 0 K with a strain rate of 10^10^ s^−1^, which is an unrealistic condition for practical deformation. Therefore, the findings of this study should be considered preliminary, and more extensive studies are required to examine the relationship between ductility and natural aging using both experimental and simulation methods.

## 6. Conclusions

This study aimed to determine the relationship between the natural aging time and ductility in Al–Si–Mg alloys. MD simulations were used to study the crack propagation behavior of pre-cracked Al containing Mg–Si clusters. The conclusions obtained from this study are as follows.

- The cracked Al containing Mg–Si clusters exhibited earlier crack opening than that without Mg–Si clusters. The Al crack tip with the Mg–Si cluster emitted Shockley partial dislocations. The strains at which the crack opened and at which dislocations began to be emitted decreased as the natural aging proceeds.
- The stress of the atoms near the crack tip for Al with larger Mg–Si clusters was more concentrated. This indicated a reduction in the effective curvature of the crack tip, which was comparable to the crack tip sharpening effect. This results from the competition between cack thinning and thickening forces applied on the solute atoms. Therefore, the crack spread more rapidly when the Mg–Si clusters were larger.
- The influence of geometrical factors was also analyzed. Owing to the crack tip sharpening effect, crack lengthening significantly contributed to decreasing the fracture toughness and tensile stress. The fracture toughness decreased as the simulation area thickness decreased; however, the magnitude of fracture toughness variation was less than that of crack lengthening.
- Considering that Mg–Si clusters grow during natural aging, it is reasonable to conclude that long-term natural aging promotes rapid crack propagation.

## Figures and Tables

**Table 1:** Values of the parameters required to calculate the LJ interatomic potential in the given MD simulation [34].

Parameter	Value
*ε_Al–Si_* (eV)	0.75
*ε_Al–Mg_* (eV)	0.3
*ε_Mg–Si_* (eV)	0.3
*σ_Al–Si_* (eV)	1.0
*σ_Al–Mg_* (eV)	1.0
*σ_Mg–Si_* (eV)	0.75
*r_c_* (Cutoff radius for the calculation of the LJ potential) (Å)	10

## Data Availability

The data underlying this article will be shared upon reasonable request to the corresponding author.
